# Association Between Serum Anion Gap and Risk of Postoperative Delirium in Patients Undergoing Gastric Surgery in ICU: A Retrospective Study From the MIMIC‐IV Database

**DOI:** 10.1155/anrp/8776973

**Published:** 2026-01-02

**Authors:** Simin Yang, Xinwei Su

**Affiliations:** ^1^ Department of Anesthesiology, Fifth Affiliated Hospital of Sun Yat-sen University, 52 Meihua East Road, Zhuhai, 519000, Guangdong, China, sysu.edu.cn; ^2^ Department of Obstetrics and Gynecology, Fifth Affiliated Hospital of Sun Yat-sen University, 52 Meihua East Road, Zhuhai, 519000, Guangdong, China, sysu.edu.cn

**Keywords:** gastric surgery, MIMIC-IV, postoperative delirium, serum anion gap

## Abstract

**Background:**

This study aimed to investigate the association between serum anion gap (AG) levels and postoperative delirium (POD) incidence in intensive care unit (ICU) patients undergoing gastric surgery.

**Methods:**

We conducted a retrospective study using data from the Medical Information Mart for Intensive Care IV (MIMIC‐IV) database. Patients who underwent gastric surgery were included to investigate the potential association between serum AG and POD risk. Restricted cubic spline (RCS) regression was used to evaluate nonlinear relationships, and receiver operating characteristic (ROC) curves were used to assess predictive performance. Subgroup and sensitivity analyses were performed to verify the reliability and consistency of the results.

**Results:**

Among the 2467 ICU patients who underwent gastric surgery, elevated serum AG levels were independently associated with increased POD risk. The RCS analysis revealed a nonlinear positive correlation between serum AG levels and the risk of POD. ROC curve analysis indicated that serum AG levels had a statistically significant but limited predictive value for POD, with an area under the curve (AUC) of 0.606 (95% CI: 0.584–0.628). Both subgroup and sensitivity analyses confirmed the robustness of these findings.

**Conclusions:**

This study establishes an independent association between serum AG and increased POD risk in ICU patients following gastric surgery, suggesting that serum AG may serve as a biomarker of physiological vulnerability for POD.

## 1. Introduction

Postoperative delirium (POD) is an acute neuropsychiatric syndrome characterized by the sudden onset of fluctuating disturbances in consciousness, attention, perception, and cognition [1]. As the most common surgical complication in older patients [2], POD typically arises from multifactorial etiologies including medication effects, metabolic derangements, and underlying medical conditions [3, 4]. This transient yet serious condition carries significant clinical consequences, being associated with prolonged hospitalization, accelerated cognitive decline, increased mortality risk, and substantial reductions in quality of life [5, 6].

Serum anion gap (AG) has gained recognition as an important prognostic biomarker for postoperative complications, with established correlations between elevated serum AG levels and adverse surgical outcomes [7–10]. In the intensive care unit (ICU) setting, serum AG measurement has become routine practice due to ubiquitous availability of blood gas analyzers. As a key metric of acid–base balance, increased serum AG typically reflects metabolic acidosis and has demonstrated prognostic value across multiple acute and chronic conditions [11, 12]. While initial studies focused on serum AG’s association with mortality in critically ill populations, more recent investigations have expanded these findings to include neurological outcomes, particularly in patients with impaired consciousness [13, 14]. These collective observations suggest that serum AG may represent a valuable predictor for POD.

The clinical significance of this potential association is underscored by Yamato et al.’s report [15], which reported a 4.5% POD incidence among gastric cancer surgery patients, highlighting the need for reliable risk stratification tools in this population. While the AG has been explored as a prognostic marker in other critical care contexts [16], to our knowledge, this is the first study to specifically investigate the association between preoperative serum AG levels and the risk of POD in ICU patients undergoing gastric surgery. To address this critical knowledge gap, we performed a comprehensive retrospective analysis utilizing the Medical Information Mart for Intensive Care IV (MIMIC‐IV) database, which provides detailed physiological and outcome data for critically ill surgical patients.

## 2. Methods

### 2.1. Data Source and Ethics Statement

The MIMIC‐IV database (Version 2.2), developed by the Computational Physiology Laboratory at the Massachusetts Institute of Technology (MIT), is an open‐access resource comprising de‐identified clinical data from patients treated at the Beth Israel Deaconess Medical Center (BIDMC) in Boston, Massachusetts, USA. This comprehensive dataset spans patient admissions from 2008 to 2019. In accordance with stringent patient privacy protocols, informed consent was not required for data collection. A member of the research team completed the Collaborative Institutional Training Initiative (CITI) program, specifically the course “Data or Specimens Only Research,” which addresses ethical considerations in human subjects research. The author Xinwei Su obtained certification (CITI Name ID: 13066813; Record ID: 61100760) and was subsequently granted access to the MIMIC‐IV database. The study design and implementation strictly adhered to established guidelines and regulatory standards, including the principles of the Declaration of Helsinki, ensuring ethical compliance throughout the research process.

### 2.2. Population Selection Criteria

**Figure 1:**
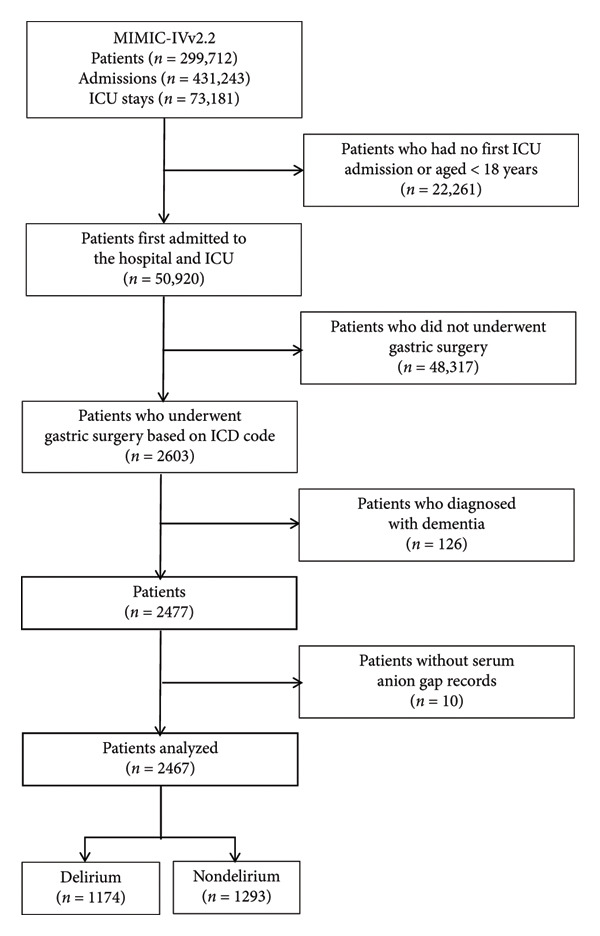
Flowchart of patient selection. Flowchart for selecting patients from the MIMIC‐IV database. ICU, intensive care unit; ICD, International Classification of Disease.

The study included patients aged 18 years or older undergoing gastric surgery, identified through International Classification of Disease (ICD) codes (Supporting Data Table S1) from the MIMIC‐IV database (Version 2.2). The exclusion criteria encompassed: (1) patients younger than 18 years at first admission, (2) ICU stays shorter than 24 h, and (3) patients lacking serum AG records. After applying these criteria, 2467 patients qualified for study inclusion. Figure 1 illustrates the patient screening process.

### 2.3. Data Extraction

Data were extracted using Structured Query Language (SQL) and Navicat Premium software (Version 16). Serum AG served as the primary study variable. The analysis included potential confounding variables, such as demographic information, comorbid conditions, laboratory results, and clinical scoring systems. The primary outcome was delirium occurrence after gastric surgery during ICU hospitalization. Delirium was diagnosed using the CAM‐ICU scoring tool, which evaluates four characteristics: (1) acute mental status changes or fluctuations; (2) inattention; (3) disorganized thinking; and (4) altered level of consciousness. Medical staff documented these characteristics on the “chartevents” form. According to the CAM‐ICU scale, a diagnosis of delirium (CAM‐ICU‐positive) requires the patient to display characteristics 1 and 2, along with either 3 or 4.

### 2.4. Statistical Analysis

To minimize data bias, variables with missing values exceeding 10% were excluded. For variables with missing data below 10%, multiple imputation was implemented. The baseline characteristics of participants were divided into two groups based on delirium presence or absence. The analysis incorporated mean (standard deviation) for normally distributed variables and median (interquartile range [17]) for non‐normally distributed continuous variables, while categorical variables were expressed as frequencies and percentages. Normality tests and quantile–quantile (QQ) plots assessed data distribution, with appropriate descriptive statistics applied accordingly. Group comparisons for normally distributed continuous variables utilized Welch’s *t*‐test or ANOVA, while non‐normally distributed variables were analyzed using the Wilcoxon rank‐sum test or Kruskal–Wallis test. For categorical data comparisons, Fisher’s exact test was applied for expected frequencies < 5, and the chi‐squared test was used otherwise. Univariate and multivariate logistic regression models examined the independent associations between serum AG levels and POD development probability. Patients were stratified into four categories based on serum AG level IQR: Q1 (5–12), Q2 (12–14), Q3 (14–17), and Q4 (17–41) to investigate the relationship between serum AG categories and outcome indicators. To address potential confounding factors, variables were systematically incorporated into the logistic regression framework through progressively adjusted models. Serum AG was analyzed as both continuous and categorical variables across four models. Model 1 remained unadjusted. Model 2 adjusted for demographic and comorbidity data. Model 3 incorporated additional laboratory parameters and disease severity scores. Model 4 included special interventions. Variance inflation factors (VIF) were calculated to assess multicollinearity, with all variables showing VIF < 5 (Supporting Data Table S2). A restricted cubic spline (RCS) regression model was used to evaluate the potential linear association between serum AG concentration and POD occurrence probability. Receiver operating characteristic (ROC) curve analysis was conducted to assess the predictive accuracy of serum AG for POD risk. Subgroup analyses examined potential effect modifiers based on various clinical factors including age, gender, and comorbidities. Sensitivity analyses assessed result robustness across different patient populations by excluding patients with cerebrovascular disease, individuals under 65 years, and analyzing the original dataset without multiple imputation [3].

In our study, all statistical analyses were conducted using R software (Version 4.2.2) and MSTATA software (https://www.mstata.com), with statistical significance set at a two‐sided *p* < 0.05.

## 3. Results

### 3.1. Sample Size and Baseline Information

The study included 2467 patients, stratified by delirium status (present/absent), as shown in Table 1. The median age was 68 years (IQR: 57–78) in the overall cohort, with no significant difference between the delirium and nondelirium groups (*p* = 0.796). Males comprised 59.22% of the population, with a slightly higher proportion in the nondelirium group (61.02% vs. 57.24%, *p* = 0.056). Patients with delirium had a higher cerebrovascular disease prevalence (40.97% vs. 24.44%, *p* < 0.001) and increased utilization of continuous renal replacement therapy (CRRT) (10.31% vs. 4.25%, *p* < 0.001), ventilation (93.87% vs. 83.37%, *p* < 0.001), benzodiazepines (53.75% vs. 45.09%, *p* < 0.001), and vasoactive medications (57.84% vs. 35.03%, *p* < 0.001). The delirium group exhibited elevated severity scores, including Simplified Acute Physiology Score II (SAPS II) (median: 38 vs. 35, *p* < 0.001) and Sequential Organ Failure Assessment (SOFA) (median: 1.00 vs. 1.00, *p* = 0.001). Laboratory values were also higher, including serum AG (median: 15.0 mmol/L vs. 14.0 mmol/L, *p* < 0.001), glucose (median: 137 mg/dL vs. 129 mg/dL, *p* < 0.001), and hemoglobin (median: 11.00 g/dL vs. 10.30 g/dL, *p* < 0.001). ICU stay (median: 13 days vs. 4 days, *p* < 0.001) and hospital stay (median: 25 days vs. 17 days, *p* < 0.001) were significantly longer in the delirium group. Other comorbidities and laboratory parameters, including chronic pulmonary disease, diabetes, and sodium levels, showed no significant differences.

**Table 1:** The baseline characteristics of participants.

Variables	Overall (*n* = 2467)	Missing data *n* (%)	Nondelirium (*n* = 1293)	Delirium (*n* = 1174)	*p* value
Age (years)	68 (57, 78)	—	68 (56, 79)	68 (57, 78)	0.796
Gender, *n* (%)		—			0.056
Male	1461 (59.22%)		789 (61.02%)	672 (57.24%)	
Female	1006 (40.78%)		504 (38.98%)	502 (42.76%)	
Cerebrovascular disease, *n* (%)		—			< 0.001
No	1670 (67.69%)		977 (75.56%)	693 (59.03%)	
Yes	797 (32.31%)		316 (24.44%)	481 (40.97%)	
Chronic pulmonary disease, *n* (%)		—			0.683
No	1850 (74.99%)		974 (75.33%)	876 (74.62%)	
Yes	617 (25.01%)		319 (24.67%)	298 (25.38%)	
Congestive heart failure, *n* (%)		—			0.117
No	1853 (75.11%)		988 (76.41%)	865 (73.68%)	
Yes	614 (24.89%)		305 (23.59%)	309 (26.32%)	
Diabetes, *n* (%)		—			0.349
No	1762 (71.42%)		934 (72.24%)	828 (70.53%)	
Yes	705 (28.58%)		359 (27.76%)	346 (29.47%)	
Myocardial infarction, *n* (%)		—			0.925
No	2101 (85.16%)		1102 (85.23%)	999 (85.09%)	
Yes	366 (14.84%)		191 (14.77%)	175 (14.91%)	
Liver disease, *n* (%)		—			0.987
No	2141 (86.79%)		1122 (86.77%)	1019 (86.80%)	
Yes	326 (13.21%)		171 (13.23%)	155 (13.20%)	
Peripheral vascular disease, *n* (%)		—			0.787
No	2169 (87.92%)		1139 (88.09%)	1030 (87.73%)	
Yes	298 (12.08%)		154 (11.91%)	144 (12.27%)	
Renal disease, *n* (%)		—			0.365
No	1998 (80.99%)		1056 (81.67%)	942 (80.24%)	
Yes	469 (19.01%)		237 (18.33%)	232 (19.76%)	
SAPS II	37 (29, 46)	—	35 (28, 44)	38 (30, 48)	< 0.001
SOFA	1.00 (0.00, 3.00)	9 (0.36%)	1.00 (0.00, 2.00)	1.00 (0.00, 3.00)	0.001
AG (mmol/L)	14.0 (12.0, 17.0)	—	14.0 (12.0, 16.0)	15.0 (13.0, 18.0)	< 0.001
BUN (mg/dL)	19 (13, 32)	1 (0.04%)	19 (13, 34)	19 (13, 30)	0.040
CA (mmol/L)	8.30 (7.80, 8.80)	2 (0.08%)	8.30 (7.70, 8.70)	8.30 (7.80, 8.80)	0.003
CL (mmol/L)	105 (101, 108)	—	105 (101, 109)	104 (100, 108)	< 0.001
CR (mg/dL)	0.90 (0.70, 1.30)	—	0.90 (0.70, 1.30)	0.90 (0.70, 1.40)	0.146
GLU (mg/dL)	133 (109, 169)	—	129 (107, 164)	137 (111, 175)	< 0.001
HB (mg/dL)	10.60 (9.00, 12.40)	1 (0.04%)	10.30 (8.80, 12.00)	11.00 (9.20, 12.80)	< 0.001
PLT (× 10^9^/L)	199 (144, 267)	1 (0.04%)	207 (148, 276)	192 (143, 256)	< 0.001
K (mmol/L)	4.10 (3.70, 4.50)	—	4.10 (3.70, 4.50)	4.10 (3.70, 4.50)	0.506
NA (mmol/L)	139.0 (136.0, 142.0)	—	139.0 (136.0, 142.0)	139.0 (136.0, 142.0)	0.606
WBC (× 10^9^/L)	11.3 (8.2, 15.3)	1 (0.04%)	11.1 (7.9, 14.8)	11.6 (8.6, 15.7)	< 0.001
CRRT		—			< 0.001
No	2291 (92.87%)		1238 (95.75%)	1053 (89.69%)	
Yes	176 (7.13%)		55 (4.25%)	121 (10.31%)	
Ventilation		—			< 0.001
No	287 (11.63%)		215 (16.63%)	72 (6.13%)	
Yes	2180 (88.37%)		1078 (83.37%)	1102 (93.87%)	
Benzodiazepines		—			< 0.001
No	1253 (50.79%)		710 (54.91%)	543 (46.25%)	
Yes	1214 (49.21%)		583 (45.09%)	631 (53.75%)	
Vasoactive drugs		—			< 0.001
No	1335 (54.11%)		840 (64.97%)	495 (42.16%)	
Yes	1132 (45.89%)		453 (35.03%)	679 (57.84%)	
Los ICU	8 (3, 18)	—	4 (2, 11)	13 (6, 22)	< 0.001
Los hospital	21 (14, 33)	—	17 (10, 27)	25 (18, 38)	< 0.001

*Note:* CA, calcium; CL, chloride; CR, creatinine; GLU, glucose; HB, hemoglobin; PLT, platelet; K, potassium; NA, sodium.

Abbreviations: AG, anion gap; BUN, blood urea nitrogen; CRRT, continuous renal replacement therapy; ICU, intensive care unit; Los, length of stay; SAPS II, Simplified Acute Physiology Score II; SOFA, Sequential Organ Failure Assessment; WBC, white blood cell.

### 3.2. Visualization of the Nonlinear Relationships by RCS

The association between serum AG and POD risk was evaluated using RCS models. In Model 1 (Figure 2(a)), Model 2 (Figure 2(b)), and Model 4 (Figure 2(d)), a nonlinear relationship emerged between serum AG and POD risk (*p* for nonlinearity < 0.05), with delirium odds increasing alongside serum AG levels. In Model 3 (Figure 2(c)), the relationship demonstrated a positive linear trend (*p* for nonlinearity = 0.055).

Figure 2Restricted cubic spline (RCS) curves showing the association between serum AG and the risk of POD in ICU patients undergoing gastric surgery. (a) Model 1 (unadjusted). (b) Model 2 (adjusted for demographic and comorbidity data). (c) Model 3 (additionally adjusted for laboratory parameters and disease severity scores). (d) Model 4 (further adjusted for special interventions). The red solid line represents the estimated OR of POD associated with serum AG levels. The red shaded area indicates the 95% CI. The bar chart at the bottom shows the distribution of patients across serum AG values.

**Figure:**
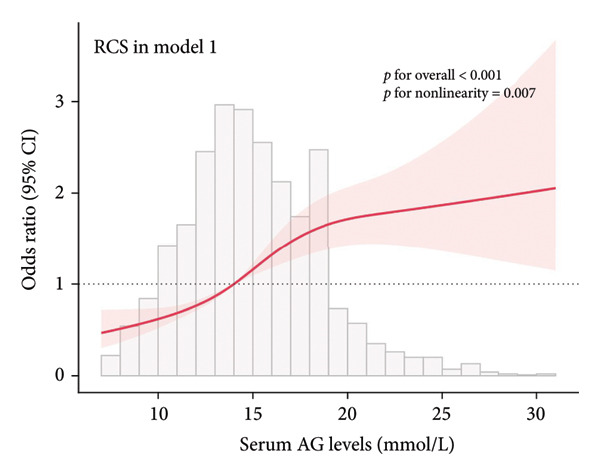
(a)

**Figure:**
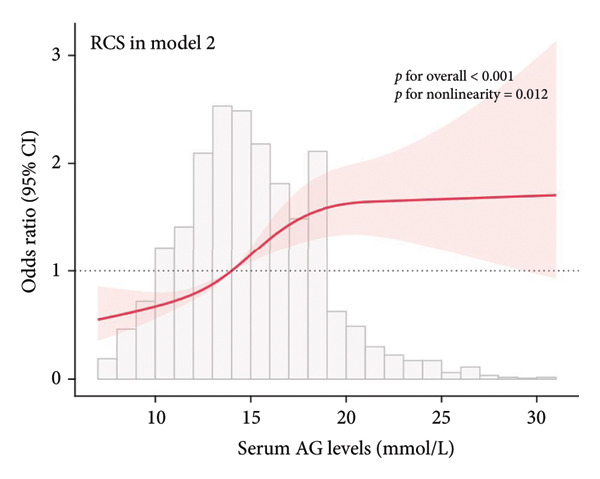
(b)

**Figure:**
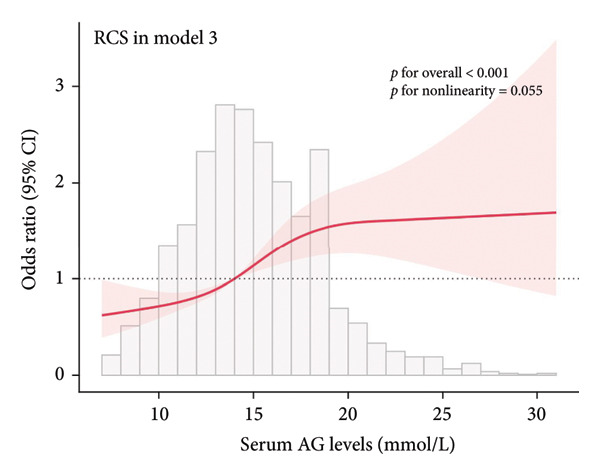
(c)

**Figure:**
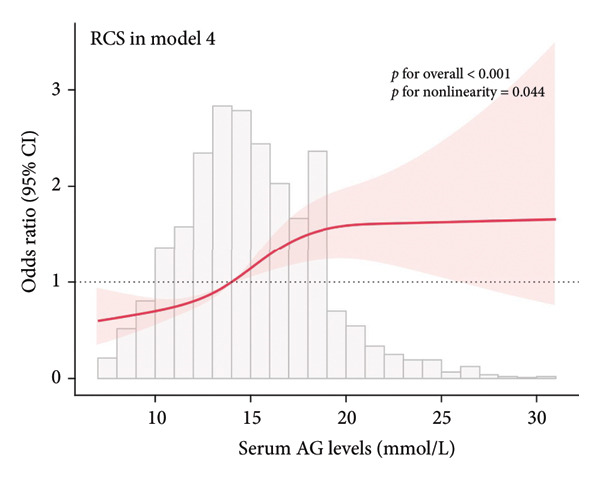
(d)

### 3.3. Predictive Value of Serum AG in POD Development Using Logistic Regression Analysis

Logistic regression analysis revealed that increasing serum AG levels consistently correlated with higher POD risk across all models in Table 2. Each unit increase in serum AG yielded an odds ratio (OR) of 1.09 (95% CI: 1.07–1.12, *p* < 0.001) in the unadjusted Model 1, 1.08 (95% CI: 1.06–1.10, *p* < 0.001) in Model 2, 1.07 (95% CI: 1.04–1.10, *p* < 0.001) in Model 3, and 1.08 (95% CI: 1.05–1.11, *p* < 0.001) in Model 4. When categorized into quartiles, higher serum AG quartiles showed progressively increased delirium risk compared to the reference quartile (Q1). Q2 demonstrated an OR of 1.49 (95% CI: 1.15–1.92, *p* = 0.002) in Model 1, showing slight decreases with adjustments in Models 2–4. Q3 exhibited an OR of 1.87 (95% CI: 1.48–2.37, *p* < 0.001) in Model 1, maintaining similar patterns in adjusted models. Q4 displayed the highest risk, with an OR of 2.96 (95% CI: 2.32–3.78, *p* < 0.001) in Model 1, remaining significant throughout all models. A significant dose–response relationship persisted, as evidenced by *p* for trend < 0.001 across all models. These findings indicate that both continuous and categorical serum AG measurements strongly correlate with increased POD risk, independent of various clinical and laboratory factors.

**Table 2:** Relationships between serum AG and the risk of POD in patients undergoing gastric surgery in the ICU.

Variable	Model 1			Model 2			Model 3			Model 4		
	OR	(95% CI)	*p*	OR	(95% CI)	*p*	OR	(95% CI)	*p*	OR	(95% CI)	*p*
AG (continuous)	1.09	(1.07, 1.12)	< 0.001	1.08	(1.06, 1.10)	< 0.001	1.07	(1.04, 1.10)	< 0.001	1.08	(1.05, 1.11)	< 0.001
AG (quintiles)												
Q1 (5, 12)	Ref	Ref		Ref	Ref		Ref	Ref		Ref	Ref	
Q2 (12, 14)	1.49	(1.15, 1.92)	0.002	1.43	(1.10, 1.86)	0.008	1.41	(1.07, 1.84)	0.013	1.44	(1.09, 1.90)	0.010
Q3 (14, 17)	1.87	(1.48, 2.37)	< 0.001	1.69	(1.32, 2.16)	< 0.001	1.56	(1.21, 2.02)	< 0.001	1.60	(1.23, 2.08)	< 0.001
Q4 (17, 41)	2.96	(2.32, 3.78)	< 0.001	2.62	(2.03, 3.38)	< 0.001	2.42	(1.82, 3.23)	< 0.001	2.50	(1.86, 3.36)	< 0.001
*p* for trend			< 0.001			< 0.001			< 0.001			< 0.001

*Note:* Model 1: No covariates were adjusted. Model 2: Adjusted for age, gender, chronic pulmonary disease, renal disease, cerebrovascular disease, myocardial infarction, peripheral vascular disease, congestive heart failure, liver disease, diabetes, SOFA, and SPAS II. Model 3: Adjusted for age, gender, chronic pulmonary disease, renal disease, cerebrovascular disease, myocardial infarction, peripheral vascular disease, congestive heart failure, liver disease, diabetes, SOFA, SPAS II, chloride, calcium, BUN, potassium, creatinine, hemoglobin, WBC, platelet, glucose, and sodium. Model 4: Adjusted for age, gender, chronic pulmonary disease, renal disease, cerebrovascular disease, myocardial infarction, peripheral vascular disease, congestive heart failure, liver disease, diabetes, SOFA, SPAS II, chloride, calcium, BUN, potassium, creatinine, hemoglobin, WBC, platelet, glucose, sodium, ventilation, CRRT, benzodiazepines, and vasoactive drugs.

Abbreviations: BUN, blood urea nitrogen; CI, confidence interval; CRRT, continuous renal replacement therapy; OR, odds ratio; SAPS II, Simplified Acute Physiology Score II; SOFA, Sequential Organ Failure Assessment; WBC, white blood cell.

### 3.4. ROC Curves

**Figure 3:**
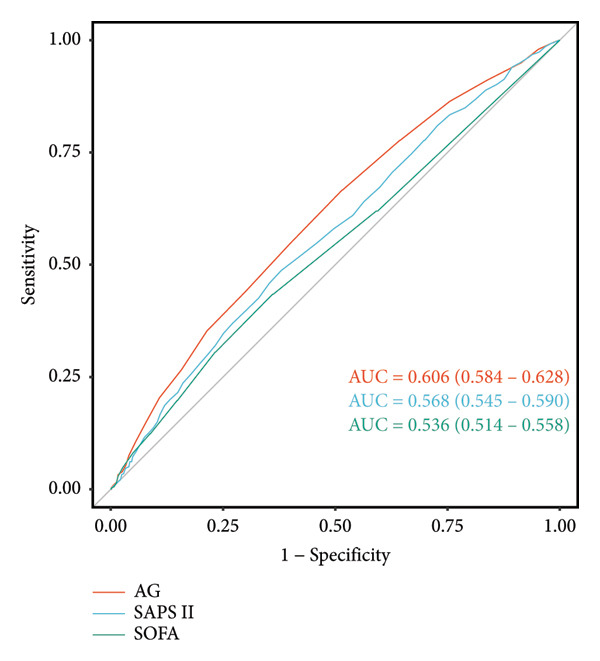
ROC curves to assess the predictive power of serum AG on the incidence of POD. AUC, area under the curve; AG, anion gap; SAPS II, Simplified Acute Physiology Score II; SOFA, Sequential Organ Failure Assessment; ROC, receiver operating characteristic; POD, postoperative delirium.

ROC curve analysis was performed to evaluate the predictive capabilities of serum AG and severity of illness scores (SAPS II and SOFA) for POD occurrence in post–gastric surgery patients. Serum AG (area under the curve [AUC] = 0.606, 95% CI: 0.584–0.628) demonstrated superior predictive performance compared to SAPS II (AUC = 0.568, 95% CI: 0.545–0.590) and SOFA (AUC = 0.536, 95% CI: 0.514–0.558), exhibiting moderate predictive accuracy (Figure 3). These findings highlight serum AG’s potential as an effective predictor of POD in gastric surgery patients.

### 3.5. Subgroup Analysis and Sensitivity Analysis

**Figure 4:**
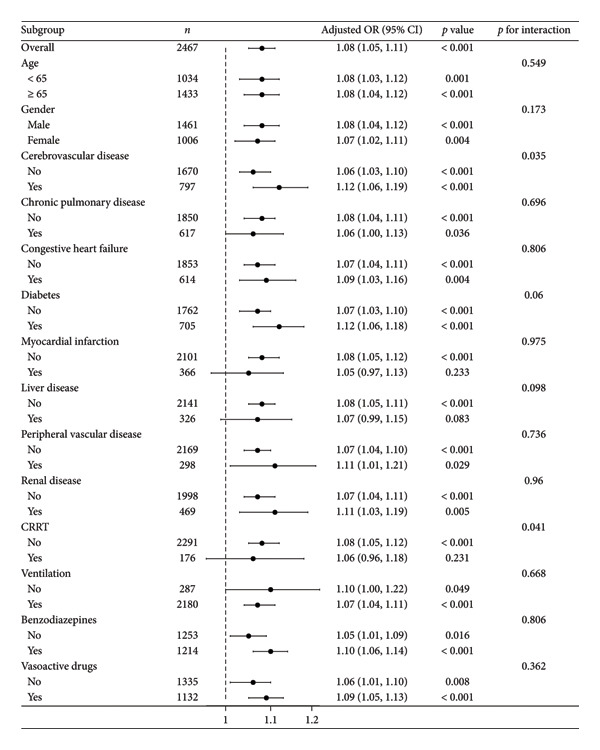
Forest plot for the relationship between serum AG and POD. Serum AG was analyzed as a continuous variable. The above models were adjusted for the variables in Model 4. In each case, the models were not adjusted for stratification variables. CRRT, continuous renal replacement therapy; AG, anion gap; POD, postoperative delirium.

Subgroup analyses examined multiple variables, including age, gender, special interventions (ventilation, CRRT, benzodiazepine use, and vasoactive drug use), and various comorbidities including cerebrovascular disease, chronic pulmonary disease, congestive heart failure, diabetes, myocardial infarction, liver disease, peripheral vascular disease, and renal disease. Results revealed no significant interactions between these subgroups (*p* for interaction > 0.05), except for cerebrovascular disease (*p* for interaction = 0.035) and CRRT (*p* for interaction = 0.041). These results suggest that the relationship between serum AG and POD risk remains consistent across most subgroups, with notable interactions in cerebrovascular disease and CRRT subgroups, as shown in Figure 4.

To ensure the robustness of our primary findings regarding the serum AG‐POD association, we conducted comprehensive sensitivity analyses. These analyses consistently demonstrated a statistically significant relationship between elevated serum AG levels and increased POD risk across all tested models (see Supporting Data Tables S3–S5). The stability of this association under varying analytical conditions strengthens the validity of our conclusions.

## 4. Discussion

Our retrospective cohort study of 2467 ICU patients undergoing gastric surgery demonstrated a significant, dose‐dependent association between elevated serum AG levels and increased POD risk. This relationship remained robust across all sensitivity analyses and predefined subgroup stratifications (age, sex, and comorbidity burden). Serum AG levels had a statistically significant but limited predictive value for POD, with an AUC of 0.606 (95% CI: 0.584–0.628). ROC analysis revealed an inherent trade‐off between the test’s sensitivity and specificity. At a threshold achieving 50% sensitivity, specificity was approximately 70%; when sensitivity was increased to 75%, specificity declined to 40%. To better evaluate clinical utility, we calculated positive and negative predictive values (PPV and NPV) across different disease prevalence scenarios. In low‐prevalence populations (e.g., 1%), the PPV was critically low (< 2%), indicating that positive results would be predominantly false positives, potentially causing unnecessary patient anxiety and resource utilization. In high‐prevalence settings (e.g., 30%), the PPV improved but remained suboptimal (35%–42%), meaning over half of positive results would still be false positives. Conversely, the NPV remained consistently high across prevalence scenarios, suggesting the test holds greater utility for ruling out disease than for confirming it. These findings indicate that this test in its current form is unsuitable for general population screening. When applied to high‐risk populations, positive results require cautious interpretation and should be integrated with other clinical indicators. Future research should focus on optimizing diagnostic thresholds or developing integrated diagnostic algorithms that incorporate this test alongside other biomarkers to enhance its predictive capability.

Despite significant improvements in gastric surgical techniques and subsequent reductions in mortality rates, postoperative neurocognitive complications—particularly delirium—continue to pose substantial clinical challenges [18]. These complications maintain high prevalence rates and significantly impair patient recovery trajectories. While the precise pathophysiology of delirium remains elusive, growing evidence implicates metabolic derangement, including acidosis, as potential contributors to its development [19, 20]. A growing body of evidence suggests that electrolyte imbalances and acid–base disturbances are associated with an increased risk of POD across various surgical contexts. For example, Li‐Hong Wang et al. reported a significant association between electrolyte disorders, including hyponatremia and hypocalcemia, and the occurrence of POD following orthopedic surgery [21]. Similarly, Zhang et al. identified electrolyte abnormalities as risk factors for POD in patients undergoing coronary artery bypass grafting [22]. Regarding acid–base imbalance, Aldemir et al. observed that metabolic acidosis was associated with a higher incidence of POD in surgical ICU patients [23]. This association has also been confirmed in a meta‐analysis by Zaal et al., which demonstrated a significant correlation between metabolic acidosis and POD in patients undergoing noncardiac surgery in the ICU [24]. To our knowledge, this is the first study to specifically investigate the association between serum AG levels and the risk of POD in ICU patients undergoing gastric surgery. Despite its statistical limitations, the primary clinical relevance of the AG lies in its role as a low‐cost, rapidly available, and routinely measured biomarker. Our study suggests that an elevated preoperative AG could serve as a simple, integrative “red flag” to identify surgical patients at higher physiological vulnerability for POD, prompting heightened clinical vigilance and the application of multidisciplinary preventive bundles.

Serum AG, calculated as the difference between unmeasured cations and anions in plasma, primarily reflects the concentration of organic acids, albumin, and phosphate [25]. As a well‐established clinical tool, serum AG serves two key functions: evaluation of acid–base disorders and differentiation of metabolic acidosis subtypes [26]. Elevated serum AG metabolic acidosis, a distinct category of metabolic disturbance, has been associated with poorer outcomes across multiple pathological states [27]. Clinical studies have consistently demonstrated correlations between increased serum AG levels and both disease severity and mortality in diverse conditions including chronic kidney disease, sepsis, acute pancreatitis, and sudden cardiac arrest [28–31].

Elevated serum AG, an indicator of metabolic acidosis, may potentiate neuroinflammation through three primary mechanistic pathways based on previous research. First, acidosis‐induced microglia activation triggers release of pro‐inflammatory mediators (e.g., nitric oxide [NO], prostaglandins, and interleukin‐1β) that amplify oxidative stress and neuroinflammatory responses [32]. Second, acidotic conditions suppress astrocytic glutamate transporters, leading to extracellular glutamate accumulation, NMDA receptor overactivation, excitotoxic calcium influx, and subsequent neuronal apoptosis [33]. Third, lactate‐mediated serum AG elevation impairs cerebrovascular function via G protein–coupled receptor 4 (GPR4) inhibition, increasing blood–brain barrier permeability and facilitating neurotoxin extravasation [34]. These pathophysiological insights suggest that serum AG may serve as a promising biomarker for POD risk in ICU patients undergoing gastric surgery. However, these proposed pathways remain hypothetical and are not directly supported by our observational data. It is critical to emphasize that AG itself is not a causal agent, but rather a biomarker reflective of broader systemic derangements. In most clinical scenarios, an elevated AG is the consequence of underlying conditions, such as hypotension, renal dysfunction, diabetes, intoxication, or other metabolic abnormalities that predispose patients to neurological compromise postoperatively, which are more plausibly the direct contributors to the development of delirium. Therefore, although our study identifies serum AG as a biomarker of POD in ICU patients undergoing gastric surgery based on the first analysis of its nonlinear association using RCSs, its clinical significance should be interpreted within the broader context of metabolic and clinical risk factors.

Our study provides the first evidence of a significant, nonlinear association between serum AG levels and POD incidence in this population, as demonstrated through RCS analysis. This study identifies serum AG as a potential biomarker associated with POD risk. Importantly, AG is a standard, routinely measured, and low‐cost biochemical parameter that is readily available in most clinical settings, including ICUs and perioperative wards. These characteristics make AG a practical and scalable tool for early risk stratification, particularly in resource‐limited environments or high‐volume surgical centers. While AG alone may not dictate clinical decisions, its inclusion in multimodal risk assessment models could enhance the identification of patients who may benefit from closer monitoring, targeted interventions, or preventive strategies against delirium. These findings support the clinical relevance of AG beyond its traditional role in metabolic monitoring, extending its utility to perioperative neurocognitive risk evaluation.

We emphasize that this is a retrospective observational study and, as such, it cannot establish causality between serum AG and POD. Our study, which is based on data from the MIMIC‐IV database, has several inherent limitations typical of retrospective designs. First, due to the retrospective nature of the data collection, inherent biases may exist, and MIMIC‐IV, which is derived primarily from a North American population, may not be fully generalizable to other regions or healthcare systems with different perioperative practices. However, we rigorously screened all eligible patients to mitigate this concern. Second, serum AG levels were measured only upon the patient’s initial admission to the ICU, and dynamic changes in serum AG were not assessed. Further research is needed to explore the potential impact of serum AG fluctuations over time. Third, we were unable to include several key variables that are known to influence POD risk, including preoperative cognitive status (e.g., MMSE and MoCA), history of delirium, anesthetic techniques, intraoperative medication use (e.g., benzodiazepines, inhalation anesthetics, and opioid administration), postoperative pain scores, sleep quality, and infection status. While we attempted to extract available proxies, certain data elements were not consistently recorded in MIMIC‐IV. These omissions may have influenced our findings and represent important avenues for future research. Additionally, although we adjusted for known confounding variables, residual or unmeasured factors may still influence the results. Given these limitations, future large‐scale, multicenter prospective studies are warranted to validate the prognostic role of serum AG in predicting the risk of POD among gastric surgery patients admitted to the ICU.

## 5. Conclusion

This study establishes an independent association between serum AG and increased POD risk in ICU patients following gastric surgery, suggesting that serum AG may function as a signal of physiological vulnerability for POD.

## Ethics Statement

The MIMIC‐IV database has been granted ethical approval by the Institutional Review Boards of the Beth Israel Deaconess Medical Center and the Massachusetts Institute of Technology.

## Consent

The requirement for informed consent was waived, given that all information in MIMIC‐IV is anonymized.

## Disclosure

All authors approved the submitted version.

## Conflicts of Interest

The authors declare no conflicts of interest.

## Author Contributions

Simin Yang and Xinwei Su designed the research. Simin Yang collected and analyzed the data and drafted the manuscript. Simin Yang and Xinwei Su revised the manuscript. All authors contributed to the article.

## Funding

The authors have nothing to report.

## Supporting Information

Supporting information is provided in a Word document containing supporting data Tables (S1–S3), listing ICD codes, multicollinearity diagnostics, and sensitivity analysis results for POD risk.

## Supporting information


**Supporting Information** Additional supporting information can be found online in the Supporting Information section.

## Data Availability

The data analyzed in this study are available from the MIMIC‐IV database.

## References

[1] Mattison M. L. P. , Delirium, Annals of Internal Medicine. (2020) 173, no. 7, ITC49–ITC64, 10.7326/AITC202010060. 33017552

[2] Yan E. , Veitch M. , Saripella A. et al., Association Between Postoperative Delirium and Adverse Outcomes in Older Surgical Patients: A Systematic Review and Meta-Analysis, Journal of Clinical Anesthesia. (2023) 90, 10.1016/j.jclinane.2023.111221. 37515876

[3] Guo P. , Ma Y. , Su W. et al., Association Between Baseline Serum Bicarbonate and the Risk of Postoperative Delirium in Patients Undergoing Cardiac Surgery in the ICU: A Retrospective Study From the MIMIC-IV Database, BMC Anesthesiology. (2024) 24, no. 1, 10.1186/s12871-024-02738-9. PMC11438213 39342157

[4] Ge Q.-Y. , Zheng C. , Song X.-B. et al., The Relationship Between the Average Infusion Rate of Propofol and the Incidence of Delirium During Invasive Mechanical Ventilation: A Retrospective Study Based on the MIMIC IV Database, CNS Neuroscience and Therapeutics. (2025) 31, no. 3, 10.1111/cns.70273. PMC11868985 40018993

[5] Huang H. , Li H. , Zhang X. et al., Association of Postoperative Delirium With Cognitive Outcomes: A Meta-Analysis, Journal of Clinical Anesthesia. (2021) 75, 10.1016/j.jclinane.2021.110496. 34482263

[6] Hoogma D. F. , Venmans E. , Al Tmimi L. et al., Postoperative Delirium and Quality of Life After Transcatheter and Surgical Aortic Valve Replacement: A Prospective Observational Study, Journal of Thoracic and Cardiovascular Surgery. (2023) 166, no. 1, 156–166.e6, 10.1016/j.jtcvs.2021.11.023. 34876283

[7] Zhao D. , Li Y. , Huang J. et al., Association of Serum Anion Gap and Risk of Long-Term Mortality in Patients Following Coronary Artery Bypass Grafting: A Propensity Score Matching Study, Journal of Cardiac Surgery. (2022) 37, no. 12, 4906–4918, 10.1111/jocs.17167. 36378900

[8] Sun X. , Lu J. , Weng W. , and Yan Q. , Association Between Anion Gap and All-Cause Mortality of Critically Ill Surgical Patients: A Retrospective Cohort Study, BMC Surgery. (2023) 23, no. 1, 10.1186/s12893-023-02137-w. PMC10413518 37559030

[9] Peng S. , Chen Q. , Ke W. , and Wu Y. , The Relationship Between Serum Anion Gap Levels and Short-Medium-and Long-Term All-Cause Mortality in ICU Patients With Congestive Heart Failure: A Retrospective Cohort Study, Acta Cardiologica. (2024) 79, no. 6, 705–719, 10.1080/00015385.2024.2371627. 38953283

[10] Wang R. , Li J. , Chen H. et al., Preoperative Albumin Corrected Anion Gap is Associated With In-Hospital and Long-Term Mortality in Patients Undergoing Coronary Artery Bypass Grafting in a Retrospective Cohort Study, Journal of Thoracic Disease. (2022) 14, no. 12, 4894–4903, 10.21037/jtd-22-1633. 36647463 PMC9840039

[11] Huang Z. , Wang S. , and Yang S. , Association Between Serum Anion Gap and Risk of in-Hospital Mortality in Patients With Acute Heart Failure, Scientific Reports. (2024) 14, no. 1, 10.1038/s41598-024-55658-6. PMC10902391 38418846

[12] Yu W. , Wen Y. , Shao Y. , Hu T. , and Wan X. , Relationship Between Anion Gap and in-Hospital Mortality in Intensive Care Patients With Liver Failure: A Retrospective Propensity Score Matching Analysis, American Journal of Translational Research. (2024) 16, no. 1, 98–108, 10.62347/uvcx1997. 38322565 PMC10839379

[13] Lu J. , Zhong L. , Yuan M. , Min J. , and Xu Y. , Association Between Serum Anion Gap and All-Cause Mortality in Patients With Acute Myocardial Infarction: A Retrospective Study Based on MIMIC-IV Database, Heliyon. (2023) 9, no. 7, 10.1016/j.heliyon.2023.e17397. PMC10395024 37539277

[14] Chen Y. , You M.-Y. , and Chu L. , Association of Albumin-Corrected Anion Gap With Severe Consciousness Disorders and Outcomes in Ischemic Stroke: A Retrospective MIMIC Analysis, Scientific Reports. (2024) 14, no. 1, 10.1038/s41598-024-76324-x. PMC11522283 39472602

[15] Yamato K. , Ikeda A. , Endo M. et al., An Association Between Cancer Type and Delirium Incidence in Japanese Elderly Patients: A Retrospective Longitudinal Study, Cancer Medicine. (2023) 12, no. 3, 2407–2416, 10.1002/cam4.5069. 35880545 PMC9939101

[16] Wang J. , Zhong H. , Chen L. et al., Association Between Anion Gap and Postoperative Delirium in Patients Undergoing Open Heart Surgery, Frontiers in Cardiovascular Medicine. (2025) 12, 10.3389/fcvm.2025.1592161. PMC12127371 40458600

[17] McDonagh F. , Carvalho J. C. A. , Abdulla S. et al., Carbetocin vs. Oxytocin at Elective Caesarean Delivery: A Double-Blind, Randomised, Controlled, Non-Inferiority Trial of Low- and High-Dose Regimens, Anaesthesia. (2022) 77, no. 8, 892–900, 10.1111/anae.15714. 35343585

[18] Tan Z. , Recent Advances in the Surgical Treatment of Advanced Gastric Cancer: A Review. Medical Science Monitor, International Medical Journal of Experimental and Clinical Research. (2019) 25, 3537–3541, 10.12659/MSM.916475, 2-s2.0-85066061237. PMC6528544 31080234

[19] Liang S. , Chau J. P. C. , Lo S. H. S. , Bai L. , Yao L. , and Choi K. C. , Validation of PREdiction of DELIRium in ICuu Patients (PRE-DELIRIC) Among Patients in Intensive Care Units: A Retrospective Cohort Study, Nursing in Critical Care. (2021) 26, no. 3, 176–182, 10.1111/nicc.12550. 32954624

[20] Poyourow C. N. , Leonberg K. , Ghajar M. , Chung M. , and Byham-Gray L. , The Role of Dietary Acid Load on Progression of Estimated Glomerular Filtration Rate Among Individuals Diagnosed With Chronic Kidney Disease, Journal of Renal Nutrition: The Official Journal of the Council on Renal Nutrition of the National Kidney Foundation. (2024) 34, no. 4, 273–282, 10.1053/j.jrn.2024.03.001. 38490515

[21] Wang L.-H. , Xu D.-J. , Wei X.-J. , Chang H.-T. , and Xu G.-H. , Electrolyte Disorders and Aging: Risk Factors for Delirium in Patients Undergoing Orthopedic Surgeries, BMC Psychiatry. (2016) 16, no. 1, 10.1186/s12888-016-1130-0, 2-s2.0-84997354369. PMC5120472 27881118

[22] Zhang W.-Y. , Wu W.-L. , Gu J.-J. et al., Risk Factors for Postoperative Delirium in Patients After Coronary Artery Bypass Grafting: A Prospective Cohort Study, Journal of Critical Care. (2015) 30, no. 3, 606–612, 10.1016/j.jcrc.2015.02.003, 2-s2.0-84928590100. 25708120

[23] Aldemir M. , Ozen S. , Kara I. H. , Sir A. , and Baç B. , Predisposing Factors for Delirium in the Surgical Intensive Care Unit, Critical Care (London, England). (2001) 5, no. 5, 265–270, 10.1186/cc1044, 2-s2.0-0034769523. 11737901 PMC83853

[24] Zaal I. J. , Devlin J. W. , Peelen L. M. , and Slooter A. J. C. , A Systematic Review of Risk Factors for Delirium in the ICU, Critical Care Medicine. (2015) 43, no. 1, 40–47, 10.1097/CCM.0000000000000625, 2-s2.0-84925683050. 25251759

[25] Azim A. , Hu B. , Gilligan S. et al., How I Evaluate a High Anion Gap Metabolic Acidosis, Clinical Journal of the American Society of Nephrology: CJASN. (2024) 19, no. 4, 525–527, 10.2215/CJN.0000000000000381. 37976122 PMC11020432

[26] Li R. , Jin X. , Ren J. et al., Relationship of Admission Serum Anion Gap and Prognosis of Critically Ill Patients: A Large Multicenter Cohort Study, Disease Markers. (2022) 2022, 10.1155/2022/5926049. PMC9771639 36569219

[27] Zhong L. , Xie B. , Ji X.-W. , and Yang X.-H. , The Association Between Albumin Corrected Anion Gap and ICU Mortality in Acute Kidney Injury Patients Requiring Continuous Renal Replacement Therapy, Internal and Emergency Medicine. (2022) 17, no. 8, 2315–2322, 10.1007/s11739-022-03093-8. 36112320 PMC9652260

[28] Asahina Y. , Sakaguchi Y. , Kajimoto S. et al., Association of Time-Updated Anion Gap With Risk of Kidney Failure in Advanced CKD: A Cohort Study, American Journal of Kidney Diseases: The Official Journal of the National Kidney Foundation. (2022) 79, no. 3, 374–382, 10.1053/j.ajkd.2021.05.022. 34280508

[29] Hu T. , Zhang Z. , and Jiang Y. , Albumin Corrected Anion Gap for Predicting In-Hospital Mortality Among Intensive Care Patients With Sepsis: A Retrospective Propensity Score Matching Analysis, Clinica Chimica Acta; International Journal of Clinical Chemistry. (2021) 521, 272–277, 10.1016/j.cca.2021.07.021. 34303712

[30] Gong F. , Zhou Q. , Gui C. , Huang S. , and Qin Z. , The Relationship Between the Serum Anion Gap and All-Cause Mortality in Acute Pancreatitis: An Analysis of the MIMIC-III Database, International Journal of General Medicine. (2021) 14, 531–538, 10.2147/IJGM.S293340. 33642873 PMC7903165

[31] Chen J. , Dai C. , Yang Y. et al., The Association Between Anion Gap and In-Hospital Mortality of Post-Cardiac Arrest Patients: A Retrospective Study, Scientific Reports. (2022) 12, no. 1, 10.1038/s41598-022-11081-3. PMC9076652 35524151

[32] Ritzel R. M. , He J. , Li Y. et al., Proton Extrusion During Oxidative Burst in Microglia Exacerbates Pathological Acidosis Following Traumatic Brain Injury, Glia. (2021) 69, no. 3, 746–764, 10.1002/glia.23926. 33090575 PMC7819364

[33] Gao J. , Duan B. , Wang D. G. et al., Coupling Between NMDA Receptor and Acid-Sensing Ion Channel Contributes to Ischemic Neuronal Death, Neuron. (2005) 48, no. 4, 635–646, 10.1016/j.neuron.2005.10.011, 2-s2.0-27844502151. 16301179

[34] Hosford P. S. , Mosienko V. , Kishi K. et al., CNS Distribution, Signalling Properties and Central Effects of G-protein Coupled Receptor 4, Neuropharmacology. (2018) 138, 381–392, 10.1016/j.neuropharm.2018.06.007, 2-s2.0-85049752818. 29894771 PMC6063991

