# Supplementary material for: Association Between Serum Anion Gap and Risk of Postoperative Delirium in Patients Undergoing Gastric Surgery in ICU: A Retrospective Study From the MIMIC‐IV Database
Source: Anesthesiol Res Pract. 2026 Jan 2;2026:8776973. doi: 10.1155/anrp/8776973 (PMC12757772; doi:10.1155/anrp/8776973)
Supplement: Supplementary file 1 — Supporting Information Additional supporting information can be found online in the Supporting Information section. [file ANRP-2026-8776973-s001.docx]

**Supplemental Data Tables**

**Supplemental Data Table S1 The ICD-9 and ICD-10 codes for identifying gastric**

|  | **ICD-9 codes** | **ICD-10 codes** |
| --- | --- | --- |
| **Stomach surgeries** | Incision and excision of stomach：430 , 4311 , 4319 , 433 , 4341 , 4342 , 4349 , 435 , 436 , 437 , 4381 , 4382 , 4389 , 4391 , 4399 Other operations on stomach： 4400 , 4401 , 4402 , 4403 , 4411 , 4412 , 4413 , 4414 , 4415 , 4419 , 4421 , 4422 , 4429 , 4431 , 4432 , 4438 , 4439 , 4440 , 4443 , 4444 , 4449 , 445 , 4461 , 4462 , 4463 , 4464 , 4465 , 4466 , 4467 , 4468 , 4469 , 4491 , 4492 , 4493 , 4494 , 4495 , 4496 , 4497 , 4498 , 4499,4441 , 4442. | Operation-Bypass ：0D16074 , 0D16079 , 0D1607A , 0D1607B , 0D1607L , 0D160J4 , 0D160J9 , 0D160JA , 0D160JB , 0D160JL , 0D160K4 , 0D160K9 , 0D160KA , 0D160KB , 0D160KL , 0D160Z4 , 0D160Z9 , 0D160ZA , 0D160ZB , 0D160ZL , 0D163J4 , 0D16474 , 0D16479 , 0D1647A , 0D1647B , 0D1647L , 0D164J4 , 0D164J9 , 0D164JA , 0D164JB , 0D164JL , 0D164K4 , 0D164K9 , 0D164KA , 0D164KB , 0D164KL , 0D164Z4 , 0D164Z9 , 0D164ZA , 0D164ZB , 0D164ZL , 0D16874 , 0D16879 , 0D1687A , 0D1687B , 0D1687L , 0D168J4 , 0D168J9 , 0D168JA , 0D168JB , 0D168JL , 0D168K4 , 0D168K9 , 0D168KA , 0D168KB , 0D168KL , 0D168Z4 , 0D168Z9 , 0D168ZA , 0D168ZB , 0D168ZL, Operation-Destruction ：0D560ZZ , 0D563ZZ , 0D564ZZ , 0D567ZZ , 0D568ZZ , 0D570ZZ , 0D573ZZ , 0D574ZZ , 0D577ZZ , 0D578ZZ , Operation-Dilation： 0D760DZ , 0D760ZZ , 0D763DZ , 0D763ZZ , 0D764DZ , 0D764ZZ , 0D767DZ , 0D767ZZ , 0D768DZ , 0D768ZZ , 0D770DZ , 0D770ZZ , 0D773DZ , 0D773ZZ , 0D774DZ , 0D774ZZ , 0D777DZ , 0D777ZZ , 0D778DZ , 0D778ZZ , Operation-Division： 0D870ZZ , 0D873ZZ , 0D874ZZ , 0D877ZZ , 0D878ZZ , Operation-Drainage： 0D9600Z , 0D960ZX , 0D960ZZ,0D9630Z , 0D963ZX , 0D963ZZ , 0D9640Z , 0D964ZX , 0D964ZZ , 0D9670Z , 0D967ZX , 0D967ZZ , 0D9680Z , 0D968ZX , 0D968ZZ , 0D9700Z , 0D970ZX , 0D970ZZ , 0D9730Z , 0D973ZX , 0D973ZZ , 0D9740Z , 0D974ZX , 0D974ZZ , 0D9770Z , 0D977ZX , 0D977ZZ , 0D9780Z , 0D978ZX , 0D978ZZ , Operation-Excision： 0DB60Z3 , 0DB60ZX , 0DB60ZZ , 0DB63Z3 , 0DB63ZX , 0DB63ZZ , 0DB64Z3 , 0DB64ZX , 0DB64ZZ , 0DB67Z3 , 0DB67ZX , 0DB67ZZ , 0DB68Z3 , 0DB68ZX , 0DB68ZZ,0DB70ZX , 0DB70ZZ , 0DB73ZX , 0DB73ZZ , 0DB74ZX , 0DB74ZZ , 0DB77ZX , 0DB77ZZ , 0DB78ZX , 0DB78ZZ Operation-Extirpation：, 0DC60ZZ , 0DC63ZZ , 0DC64ZZ , 0DC67ZZ , 0DC68ZZ , 0DC70ZZ , 0DC73ZZ , 0DC74ZZ , 0DC77ZZ , 0DC78ZZ ,Operation-Extraction ： 0DD63ZX , 0DD64ZX , 0DD68ZX , 0DD73ZX , 0DD74ZX , 0DD78ZX , Operation-Fragmentation ： 0DF60ZZ , 0DF63ZZ , 0DF64ZZ , 0DF67ZZ , 0DF68ZZ , 0DF6XZZ , Operation-Insertion： 0DH602Z , 0DH603Z , 0DH60DZ , 0DH60MZ , 0DH60UZ , 0DH60YZ , 0DH632Z , 0DH633Z , 0DH63DZ , 0DH63MZ , 0DH63UZ , 0DH63YZ , 0DH642Z , 0DH643Z , 0DH64DZ , 0DH64MZ , 0DH64UZ , 0DH64YZ , 0DH672Z , 0DH673Z , 0DH67DZ , 0DH67UZ , 0DH67YZ , 0DH682Z , 0DH683Z , 0DH68DZ , 0DH68UZ , 0DH68YZ , Operation-Inspection： 0DJ60ZZ , 0DJ63ZZ , 0DJ64ZZ , 0DJ67ZZ , 0DJ68ZZ , 0DJ6XZZ , Operation-Occlusion： 0DL60CZ , 0DL60DZ , 0DL60ZZ , 0DL63CZ , 0DL63DZ , 0DL63ZZ , 0DL64CZ , 0DL64DZ , 0DL64ZZ , 0DL67DZ , 0DL67ZZ , 0DL68DZ , 0DL68ZZ , 0DL70CZ , 0DL70DZ , 0DL70ZZ , 0DL73CZ , 0DL73DZ , 0DL73ZZ , 0DL74CZ , 0DL74DZ , 0DL74ZZ , 0DL77DZ , 0DL77ZZ , 0DL78DZ , 0DL78ZZ, Operation-Revision：0DW600Z , 0DW602Z , 0DW603Z , 0DW607Z , 0DW60CZ , 0DW60DZ , 0DW60JZ , 0DW60KZ , 0DW60MZ , 0DW60UZ , 0DW60YZ , 0DW630Z , 0DW632Z , 0DW633Z , 0DW637Z , 0DW63CZ , 0DW63DZ , 0DW63JZ , 0DW63KZ , 0DW63MZ , 0DW63UZ , 0DW63YZ , 0DW640Z , 0DW642Z , 0DW643Z , 0DW647Z , 0DW64CZ , 0DW64DZ , 0DW64JZ , 0DW64KZ , 0DW64MZ , 0DW64UZ , 0DW64YZ , 0DW670Z , 0DW672Z , 0DW673Z , 0DW677Z , 0DW67CZ , 0DW67DZ , 0DW67JZ , 0DW67KZ , 0DW67UZ , 0DW67YZ , 0DW680Z , 0DW682Z , 0DW683Z , 0DW687Z , 0DW68CZ , 0DW68DZ , 0DW68JZ , 0DW68KZ , 0DW68UZ , 0DW68YZ , 0DW6X0Z , 0DW6X2Z , 0DW6X3Z , 0DW6X7Z , 0DW6XCZ , 0DW6XDZ , 0DW6XJZ , 0DW6XKZ , 0DW6XUZ , Operation-Transfer： 0DX60Z5 , 0DX64Z5 |

**Supplemental Data Table S2 Variance Inflation Factor and Tolerance**

| Term | VIF | SE factor | Tolerance |
| --- | --- | --- | --- |
| AG | 1.522429 | 1.233868 | 0.6568449 |
| Age | 1.560591 | 1.249236 | 0.6407828 |
| Gender | 1.102421 | 1.049962 | 0.9070949 |
| Cerebrovascular Disease | 1.219771 | 1.104433 | 0.8198258 |
| Chronic Pulmonary Disease | 1.099551 | 1.048595 | 0.9094619 |
| Congestive Heart Failure | 1.279714 | 1.131244 | 0.7814247 |
| Diabetes | 1.252154 | 1.118997 | 0.7986235 |
| Myocardial Infarct | 1.116859 | 1.056816 | 0.8953679 |
| Liver Disease | 1.144072 | 1.069613 | 0.8740712 |
| Peripheral Vascular Disease | 1.070833 | 1.034811 | 0.9338520 |
| Renal Disease | 1.552546 | 1.246012 | 0.6441032 |
| SAPSII | 1.736610 | 1.317805 | 0.5758344 |
| SOFA | 1.334890 | 1.155374 | 0.7491253 |
| BUN | 2.309014 | 1.519544 | 0.4330852 |
| Calcium | 1.313907 | 1.146258 | 0.7610888 |
| Chloride | 3.185325 | 1.784748 | 0.3139397 |
| Creatinine | 2.546860 | 1.595889 | 0.3926403 |
| Glucose | 1.223480 | 1.106110 | 0.8173410 |
| Hemoglobin | 1.362731 | 1.167361 | 0.7338208 |
| Platelet | 1.178945 | 1.085792 | 0.8482162 |
| Potassium | 1.256716 | 1.121033 | 0.7957250 |
| Sodium | 2.945867 | 1.716353 | 0.3394586 |
| WBC | 1.092199 | 1.045083 | 0.9155839 |
| CRRT | 1.282144 | 1.132318 | 0.7799439 |
| Ventilation | 1.087653 | 1.042906 | 0.9194107 |
| Benzodiazepines | 1.154130 | 1.074304 | 0.8664535 |
| Vasoactive Drugs | 1.295118 | 1.138032 | 0.7721307 |

**Supplemental Data Table S3 Relationships between serum AG and t the risk of POD in patients undergoing gastric surgery in the ICU after excluding patients with cerebrovascular disease.**

| **variable** | **Model 1** | | | **Model 2** | | | **Model 3** | | | **Model 4** | | |
| --- | --- | --- | --- | --- | --- | --- | --- | --- | --- | --- | --- | --- |
|  | **OR** | **(95% CI)** | ***p*** | **OR** | **(95% CI)** | ***p*** | **OR** | **(95% CI)** | ***p*** | **OR** | **(95% CI)** | ***p*** |
| AG(Continuous) | 1.08 | (1.05, 1.10) | <0.001 | 1.06 | (1.04, 1.09) | <0.001 | 1.06 | (1.03, 1.09) | <0.001 | 1.06 | (1.03, 1.10) | <0.001 |
| AG(Quintiles) |  |  |  |  |  |  |  |  |  |  |  |  |
| Q1 (5,12) | ref | ref |  | ref | ref |  | ref | ref |  | ref | ref |  |
| Q2 (12,14) | 1.47 | (1.08, 2.01) | 0.013 | 1.56 | (1.14, 2.14) | 0.006 | 1.49 | (1.08, 2.07) | 0.015 | 1.54 | (1.10, 2.16) | 0.013 |
| Q3 (14,17) | 1.58 | (1.19, 2.12) | 0.002 | 1.59 | (1.19, 2.14) | 0.002 | 1.45 | (1.07, 1.99) | 0.018 | 1.47 | (1.07, 2.03) | 0.019 |
| Q4 (17,41) | 2.60 | (1.95, 3.47) | <0.001 | 2.39 | (1.77, 3.24) | <0.001 | 2.17 | (1.55, 3.05) | <0.001 | 2.22 | (1.56, 3.17) | <0.001 |
| *p* for trend |  |  | <0.001 |  |  | <0.001 |  |  | <0.001 |  |  | <0.001 |

OR, Odds Ratio; CI, Confidence Interval; SOFA, sequential organ failure assessment; SAPS II, Simplified Acute Physiology Score II; BUN, blood urea nitrogen; WBC, white blood cell; CRRT, continuous renal replacement therapy.

Model 1: no covariates were adjusted.

Model 2: adjusted for age, gender, chronic pulmonary disease, renal disease, cerebrovascular disease, myocardial infarct, peripheral vascular disease, congestive heart failure, liver disease, diabetes，SOFA, and SPAS II.

Model 3: adjusted for age, gender, chronic pulmonary disease, renal disease, cerebrovascular disease, myocardial infarct, peripheral vascular disease, congestive heart failure, liver disease, diabetes, SOFA, SPAS II, chloride, calcium, BUN, potassium, creatinine, hemoglobin, WBC, platelet, glucose, and sodium.

Model 4: adjusted for age, gender, chronic pulmonary disease, renal disease, cerebrovascular disease, myocardial infarct, peripheral vascular disease, congestive heart failure, liver disease, diabetes, SOFA, SPAS II, chloride, calcium, BUN, potassium, creatinine, hemoglobin, WBC, platelet, glucose, sodium, ventilation, CRRT, benzodiazepines, and vasoactive drugs.

**Supplemental Data Table S4 Relationships between serum AG and t the risk of POD in patients undergoing gastric surgery in the ICU after excluding patients under 65 years**

| **variable** | **Model 1** | | | **Model 2** | | | **Model 3** | | | **Model 4** | | |
| --- | --- | --- | --- | --- | --- | --- | --- | --- | --- | --- | --- | --- |
|  | **OR** | **(95% CI)** | ***p*** | **OR** | **(95% CI)** | ***p*** | **OR** | **(95% CI)** | ***p*** | **OR** | **(95% CI)** | ***p*** |
| AG(Continuous) | 1.09 | (1.06, 1.12) | <0.001 | 1.07 | (1.04, 1.11) | <0.001 | 1.07 | (1.03, 1.11) | <0.001 | 1.08 | (1.04, 1.12) | <0.001 |
| AG(Quintiles) |  |  |  |  |  |  |  |  |  |  |  |  |
| Q1 (5,12) | ref | ref |  | ref | ref |  | ref | ref |  | ref | ref |  |
| Q2 (12,14) | 1.66 | (1.19, 2.33) | 0.003 | 1.56 | (1.10, 2.21) | 0.013 | 1.55 | (1.08, 2.23) | 0.018 | 1.59 | (1.10, 2.32) | 0.014 |
| Q3 (14,17) | 2.03 | (1.48, 2.81) | <0.001 | 1.91 | (1.37, 2.67) | <0.001 | 1.79 | (1.26, 2.55) | 0.001 | 1.95 | (1.36, 2.81) | <0.001 |
| Q4 (17,41) | 3.10 | (2.24, 4.30) | <0.001 | 2.76 | (1.96, 3.90) | <0.001 | 2.70 | (1.83, 3.99) | <0.001 | 2.93 | (1.95, 4.40) | <0.001 |
| *p* for trend |  |  | <0.001 |  |  | <0.001 |  |  | <0.001 |  |  | <0.001 |

OR, Odds Ratio; CI, Confidence Interval; SOFA, sequential organ failure assessment; SAPS II, Simplified Acute Physiology Score II; BUN, blood urea nitrogen; WBC, white blood cell; CRRT, continuous renal replacement therapy.

Model 1: no covariates were adjusted.

Model 2: adjusted for age, gender, chronic pulmonary disease, renal disease, cerebrovascular disease, myocardial infarct, peripheral vascular disease, congestive heart failure, liver disease, diabetes，SOFA, and SPAS II.
Model 3: adjusted for age, gender, chronic pulmonary disease, renal disease, cerebrovascular disease, myocardial infarct, peripheral vascular disease, congestive heart failure, liver disease, diabetes, SOFA, SPAS II, chloride, calcium, BUN, potassium, creatinine, hemoglobin, WBC, platelet, glucose, and sodium.

Model 4: adjusted for age, gender, chronic pulmonary disease, renal disease, cerebrovascular disease, myocardial infarct, peripheral vascular disease, congestive heart failure, liver disease, diabetes, SOFA, SPAS II, chloride, calcium, BUN, potassium, creatinine, hemoglobin, WBC, platelet, glucose, sodium, ventilation, CRRT, benzodiazepines, and vasoactive drugs.

**Supplemental Data Table S5 Relationships between serum AG and t the risk of POD in patients undergoing gastric surgery in the ICU in the original data before multiple interpolation.**

| **variable** | **Model 1** | | | **Model 2** | | | **Model 3** | | | **Model 4** | | |
| --- | --- | --- | --- | --- | --- | --- | --- | --- | --- | --- | --- | --- |
|  | **OR** | **(95% CI)** | ***p*** | **OR** | **(95% CI)** | ***p*** | **OR** | **(95% CI)** | ***p*** | **OR** | **(95% CI)** | ***p*** |
| AG(Continuous) | 1.09 | (1.07, 1.12) | <0.001 | 1.08 | (1.06, 1.10) | <0.001 | 1.07 | (1.05, 1.10) | <0.001 | 1.08 | (1.05, 1.11) | <0.001 |
| AG(Quintiles) |  |  |  |  |  |  |  |  |  |  |  |  |
| Q1 (5,12) | ref | ref |  | ref | ref |  | ref | ref |  | ref | ref |  |
| Q2 (12,14) | 1.49 | (1.15, 1.92) | 0.002 | 1.44 | (1.11, 1.87) | 0.007 | 1.43 | (1.09, 1.88) | 0.009 | 1.47 | (1.11, 1.93) | 0.007 |
| Q3 (14,17) | 1.87 | (1.48, 2.37) | <0.001 | 1.71 | (1.34, 2.19) | <0.001 | 1.58 | (1.23, 2.05) | <0.001 | 1.62 | (1.25, 2.12) | <0.001 |
| Q4 (17,41) | 2.96 | (2.32, 3.78) | <0.001 | 2.65 | (2.06, 3.43) | <0.001 | 2.47 | (1.86, 3.30) | <0.001 | 2.55 | (1.90, 3.44) | <0.001 |
| *p* for trend |  |  | <0.001 |  |  | <0.001 |  |  | <0.001 |  |  | <0.001 |

OR, Odds Ratio; CI, Confidence Interval; SOFA, sequential organ failure assessment; SAPS II, Simplified Acute Physiology Score II; BUN, blood urea nitrogen; WBC, white blood cell; CRRT, continuous renal replacement therapy.

Model 1: no covariates were adjusted.

Model 2: adjusted for age, gender, chronic pulmonary disease, renal disease, cerebrovascular disease, myocardial infarct,

peripheral vascular disease, congestive heart failure, liver disease, diabetes，SOFA, and SPAS II.

Model 3: adjusted for age, gender, chronic pulmonary disease, renal disease, cerebrovascular disease, myocardial infarct, peripheral vascular disease, congestive heart failure, liver disease, diabetes, SOFA, SPAS II, chloride, calcium, BUN, potassium, creatinine, hemoglobin, WBC, platelet, glucose, and sodium.

Model 4: adjusted for age, gender, chronic pulmonary disease, renal disease, cerebrovascular disease, myocardial infarct, peripheral vascular disease, congestive heart failure, liver disease, diabetes, SOFA, SPAS II, chloride, calcium, BUN, potassium, creatinine, hemoglobin, WBC, platelet, glucose, sodium, ventilation, CRRT, benzodiazepines, and vasoactive drugs.
